# Angiomatous Meningioma With Postictal Hemiparesis: Clinical Presentation and Surgical Challenges in a Rare Case

**DOI:** 10.7759/cureus.97553

**Published:** 2025-11-23

**Authors:** Idalberto Luis Fernandez Eng, Alpha Singuepire, Yelka Matos Furones, Elizabeth Blanco Espinosa, Ruben E Diaz Samada

**Affiliations:** 1 Emergency, Hospital Universitario de la Ribera, Valencia, ESP; 2 Neurological Surgery, Hôpital Nianankoro Fomba de Segou, Segou, MLI; 3 North Georgia Clinical Research/Neurology, Alcanza Clinical Research, Woodstock, USA; 4 General Practice, Ceda Orthopedic Group, Miami, USA; 5 Surgery, Hospital Universitario Arnaldo Milian Castro, Santa Clara, CUB; 6 Internal Medicine, Saturnino Lora Clinical-Surgical Teaching Hospital, Santiago de Cuba, CUB

**Keywords:** angiomatous meningioma, case report, craniotomy, histopathology, meningioma, neuroimaging, seizures, simpson grade ii resection

## Abstract

Angiomatous meningioma (AM) is a rare histological variant of meningioma characterized by prominent vascular proliferation, often posing intraoperative hemostatic challenges. We report the case of a 42-year-old man presenting with recurrent generalized tonic-clonic seizures and postictal right hemiparesis. CT imaging revealed a large, enhancing left parietal mass with marked perilesional edema and mass effect. In this rural setting, MRI and cerebral angiography were unavailable, requiring surgical planning to rely solely on CT findings. The patient underwent a wide left parietal craniotomy, where a highly vascular lesion was encountered. Early devascularization of dural feeders, stepwise debulking, and meticulous microsurgical dissection achieved gross total resection (Simpson grade II) with effective hemostasis. Histopathology confirmed AM. Postoperatively, the patient showed progressive neurological improvement, remained seizure-free, and was discharged on oral levetiracetam; follow-up imaging confirmed complete resection. This case underscores the need to consider AM in patients with seizure-related hemiparesis and demonstrates that, even without advanced vascular imaging, careful CT-based planning and precise microsurgical technique can yield excellent outcomes in resource-limited environments.

## Introduction

Meningiomas are the most common primary intracranial tumors, arising from the meninges - the protective membranes surrounding the brain. They comprise a heterogeneous group with multiple histological variants. Among these, angiomatous meningioma (AM) is a rare subtype, accounting for approximately 2.1% of all meningiomas, and is characterized by a highly vascular composition in which more than half of the tumor volume consists of blood vessels [1].

According to the 2021 World Health Organization (WHO) classification of central nervous system tumors, meningiomas are divided into 15 histological subtypes, with AM recognized as a distinct but uncommon variant [2,3]. Radiologically, AM often mimics other meningioma subtypes, making accurate preoperative differentiation challenging. AM typically presents with a slow onset of progressively developing symptoms. The primary manifestations arise from the tumor compressing adjacent structures, leading to seizures and compression-related effects such as motor deficits, headaches, and nausea; radicular pain is also a common symptom reported by patients [1].

Certain imaging features, such as marked peritumoral edema and prominent vascularity, may suggest the diagnosis; however, histopathological and immunohistochemical confirmation remains essential [3]. Immunohistochemistry typically demonstrates positivity for epithelial membrane antigen (EMA) and progesterone receptors [4].

Surgical resection remains the mainstay of treatment. However, the rich vascular supply of AM poses a significant intraoperative bleeding risk and increases the likelihood of incomplete resection, which in turn may predispose to recurrence. Despite these challenges, AMs are usually classified as WHO grade I tumors and are associated with a favorable prognosis when gross total resection is achieved [1].

Here, we report the case of a 42-year-old man who presented with generalized tonic-clonic seizures followed by right-sided postictal hemiparesis, later found to harbor an AM. This case not only illustrates the diagnostic and surgical challenges posed by this rare subtype but also underscores the feasibility of achieving a successful outcome using CT-based planning alone in a resource-limited setting without access to MRI or angiographic studies. By documenting this experience, we aim to contribute to the limited literature on the management of highly vascular intracranial tumors under constrained healthcare conditions.

## Case presentation

A 42-year-old African male, a high school mathematics teacher and chronic smoker (20 pack-years) with no other comorbidities, presented to the neurosurgical outpatient clinic in August 2025 after his third generalized tonic-clonic seizure in three years. Each episode was followed by transient right hemiparesis. Neurological examination revealed a grade 4/5 right hemiparesis without sensory deficits; cranial nerves and vital signs were within normal limits.

The patient reported prior seizures in 2022 and 2024, for which he had presented to the emergency department and was treated for presumptive malaria. No neuroimaging studies were performed at those visits. At presentation, he was started on carbamazepine 200 mg twice daily, omeprazole 40 mg daily, methylprednisolone 4 mg daily, and acetaminophen 500 mg three times daily.

Initial laboratory studies revealed mild neutrophilia (absolute neutrophil count: 7.98 × 10⁹/L; reference range: 1.20-7.50 × 10⁹/L) with relative lymphopenia (absolute lymphocyte count: 0.61 × 10⁹/L; reference range: 1.50-4.00 × 10⁹/L). Red blood cell count (4.54 × 10¹²/L; reference range: 4.00-5.40 × 10¹²/L), hemoglobin (13.3 g/dL; reference range: 12.0-16.0 g/dL), platelet count (240 × 10⁹/L; reference range: 150-400 × 10⁹/L), and total white blood cell count (9.08 × 10⁹/L; reference range: 4.0-10.0 × 10⁹/L) were within normal limits.

**Figure 1:**
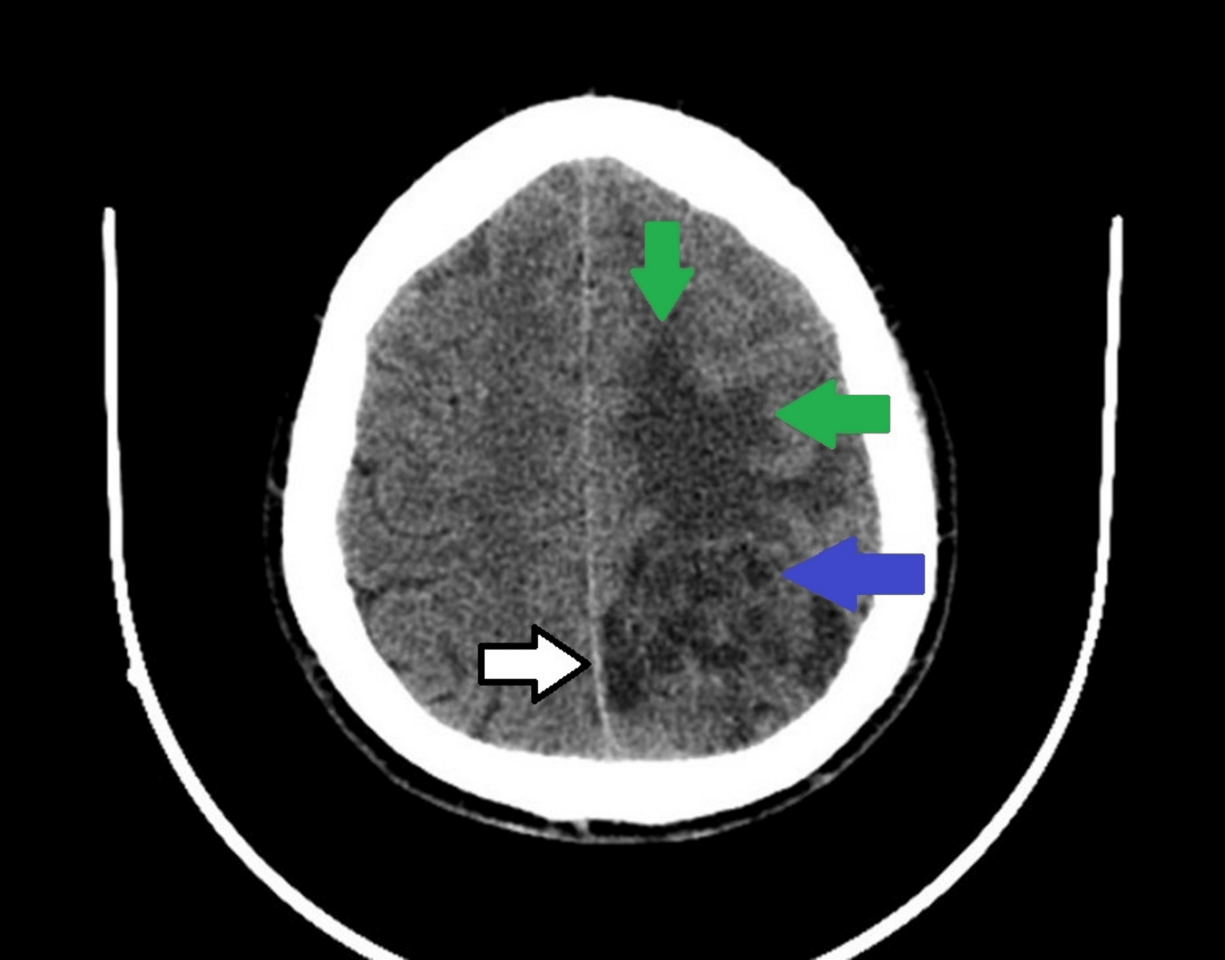
Non-contrast head CT scan showing a left parietal mass (blue arrow) with marked perilesional edema (green arrows) and mild rightward midline shift (white arrow with a black outline).

**Figure 2:**
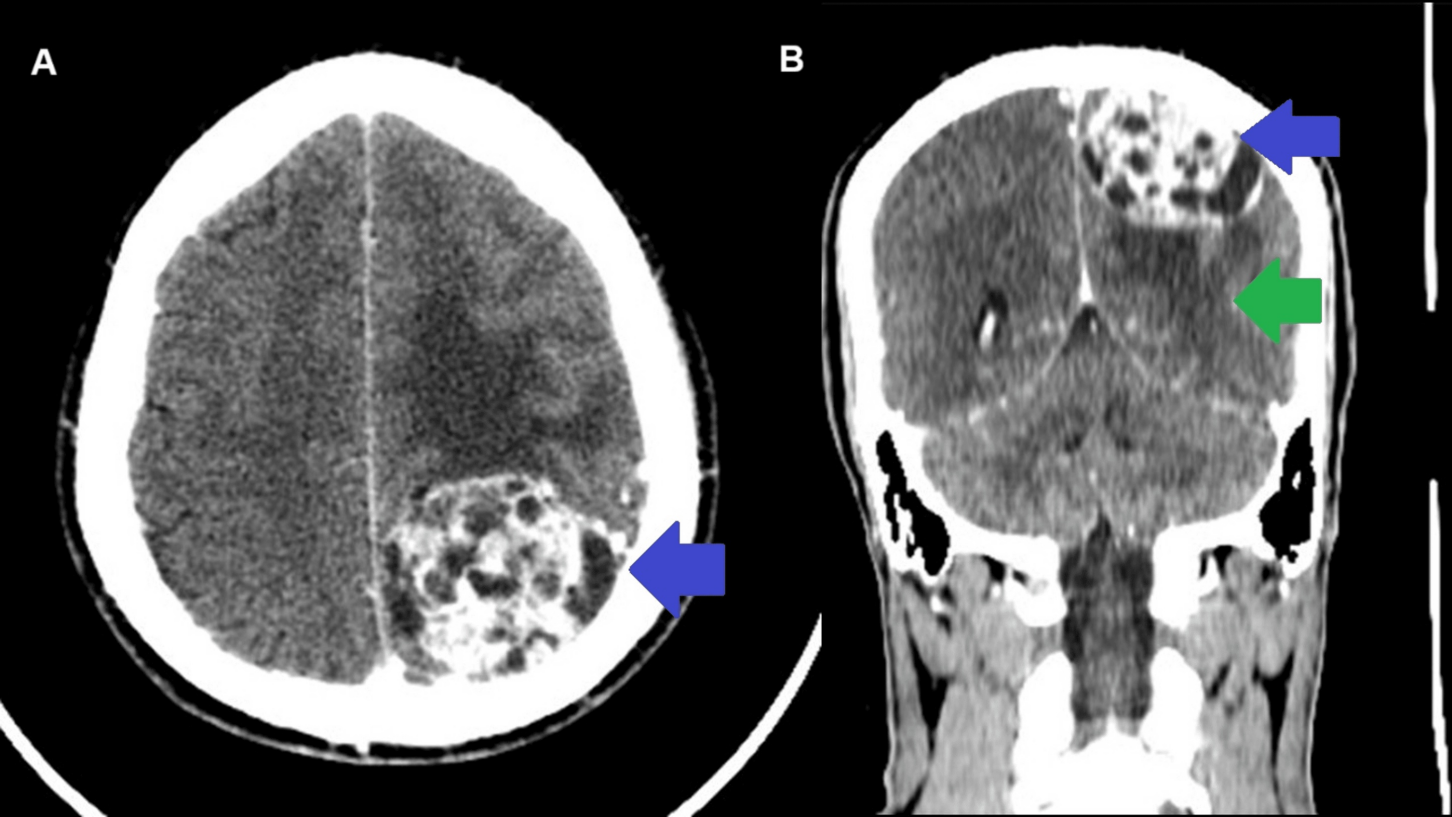
Contrast-enhanced head CT scans. (A) Axial and (B) coronal views showing a 44 × 44 mm heterogeneous hypodense parietal lesion with intense enhancement (blue arrows), causing mass effect with sulcal effacement, perilesional edema, and compression of the left lateral ventricle (green arrow).

A non-contrast CT scan of the head showed a hypodense left parietal mass with significant perilesional edema, compressing the roof, lateral wall, and posterior horn of the left lateral ventricle, and displacing the cingulate gyrus beneath the falx (Figure 1). No adjacent bone hyperostosis was observed. Contrast-enhanced CT with Omnipaque revealed a 44 × 44 mm heterogeneous hypodense parietal lesion with intense enhancement, causing sulcal effacement, left lateral ventricle compression, and mild rightward midline shift, but no calcifications, hemorrhage, necrosis, or bone involvement (Figure 2).

Given the unavailability of MRI and cerebral angiography at this rural hospital, a presumptive diagnosis of a low-grade glial tumor was made based on CT findings. Although referral to a more specialized center was considered, the patient’s socioeconomic circumstances prevented transfer. This lack of access to advanced preoperative imaging posed a significant challenge for surgical planning, leaving the team without detailed information on the lesion’s vascular architecture.

**Figure 3:**
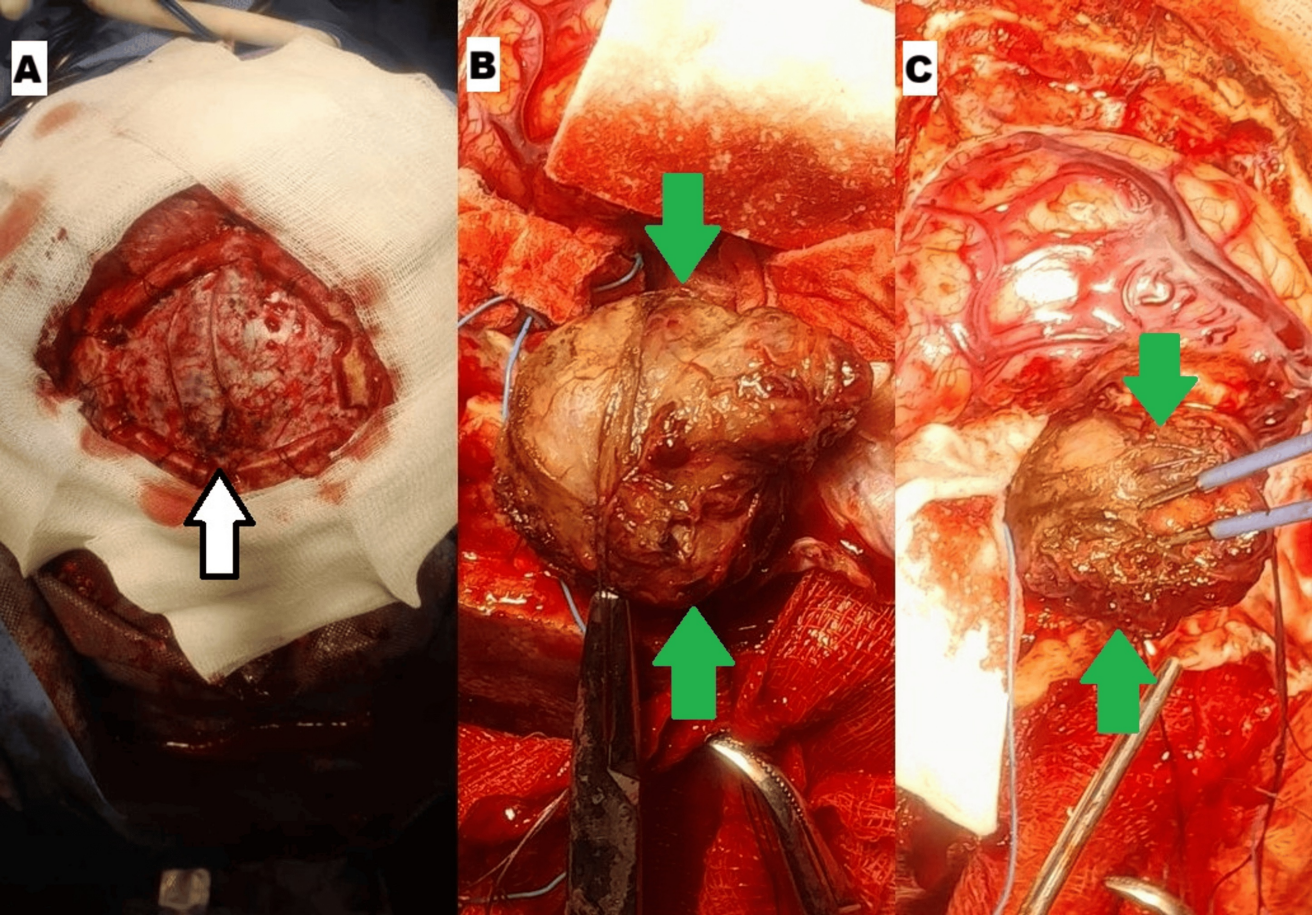
(A) Craniotomy site (white arrow with black border). (B, C) Well-defined and highly vascularized lesion (green arrows).

**Figure 4:**
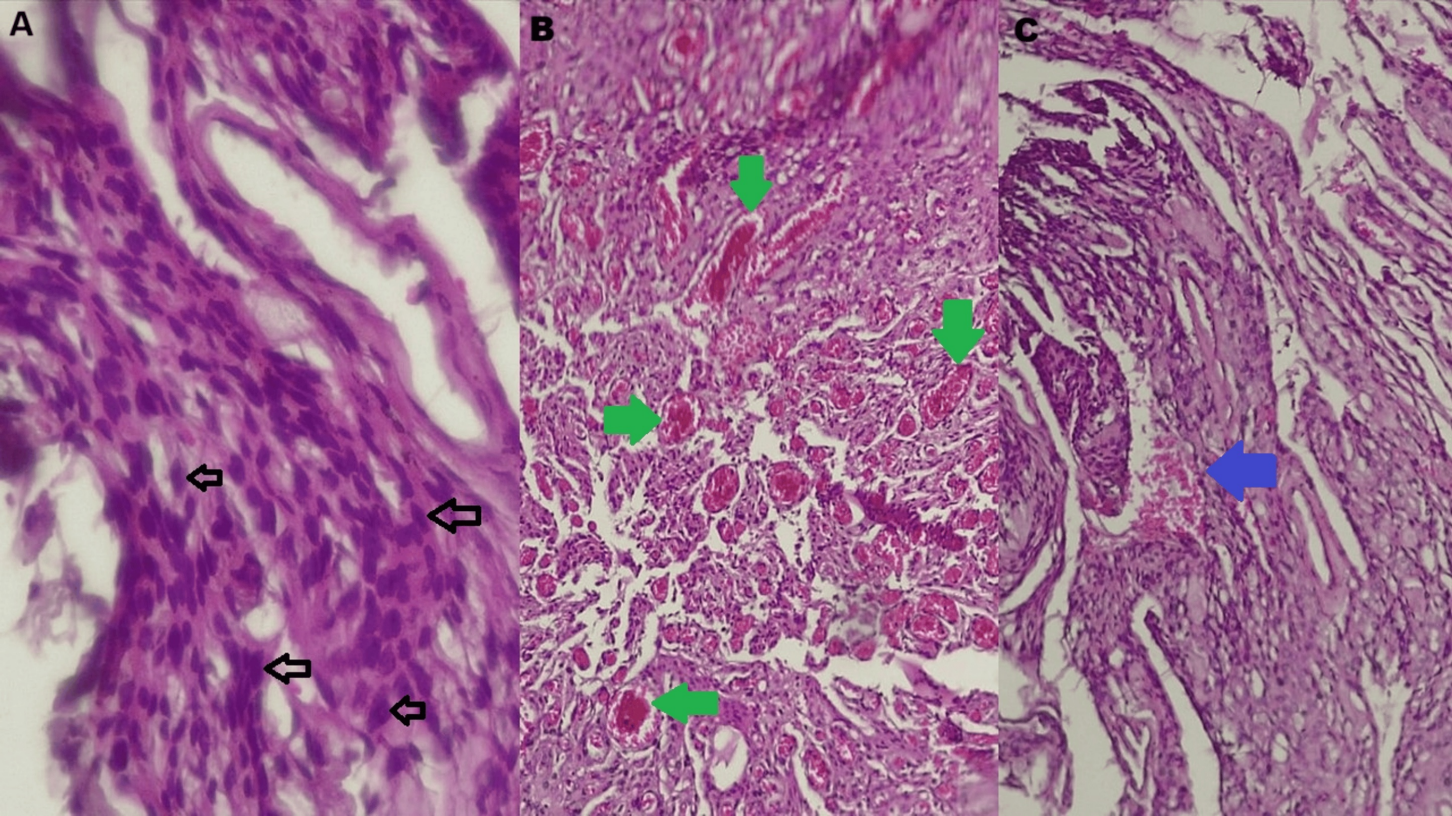
Light microscopy with hematoxylin and eosin images. (A) Abundant meningothelial cells (black arrows), (B) prominent neovascular proliferation (green arrows), and (C) areas of hemorrhage (blue arrow).

The patient underwent a conventional left parietal craniotomy under general anesthesia with the head secured in a three-point headrest. After dural opening, a highly vascular, well-circumscribed lesion was encountered (Figure 3). Careful dissection and meticulous hemostasis allowed for gross total resection, achieving Simpson grade II excision. The procedure lasted four hours and 40 minutes, with prolonged operative time due to persistent low-volume bleeding from the tumor. Histopathological examination confirmed the intraoperative impression, revealing a fasciculated proliferation of meningothelial cells without atypia, with abundant vascular channels, hemorrhagic areas, and no features of malignancy. These findings established the definitive diagnosis of AM (Figure 4).

**Figure 5:**
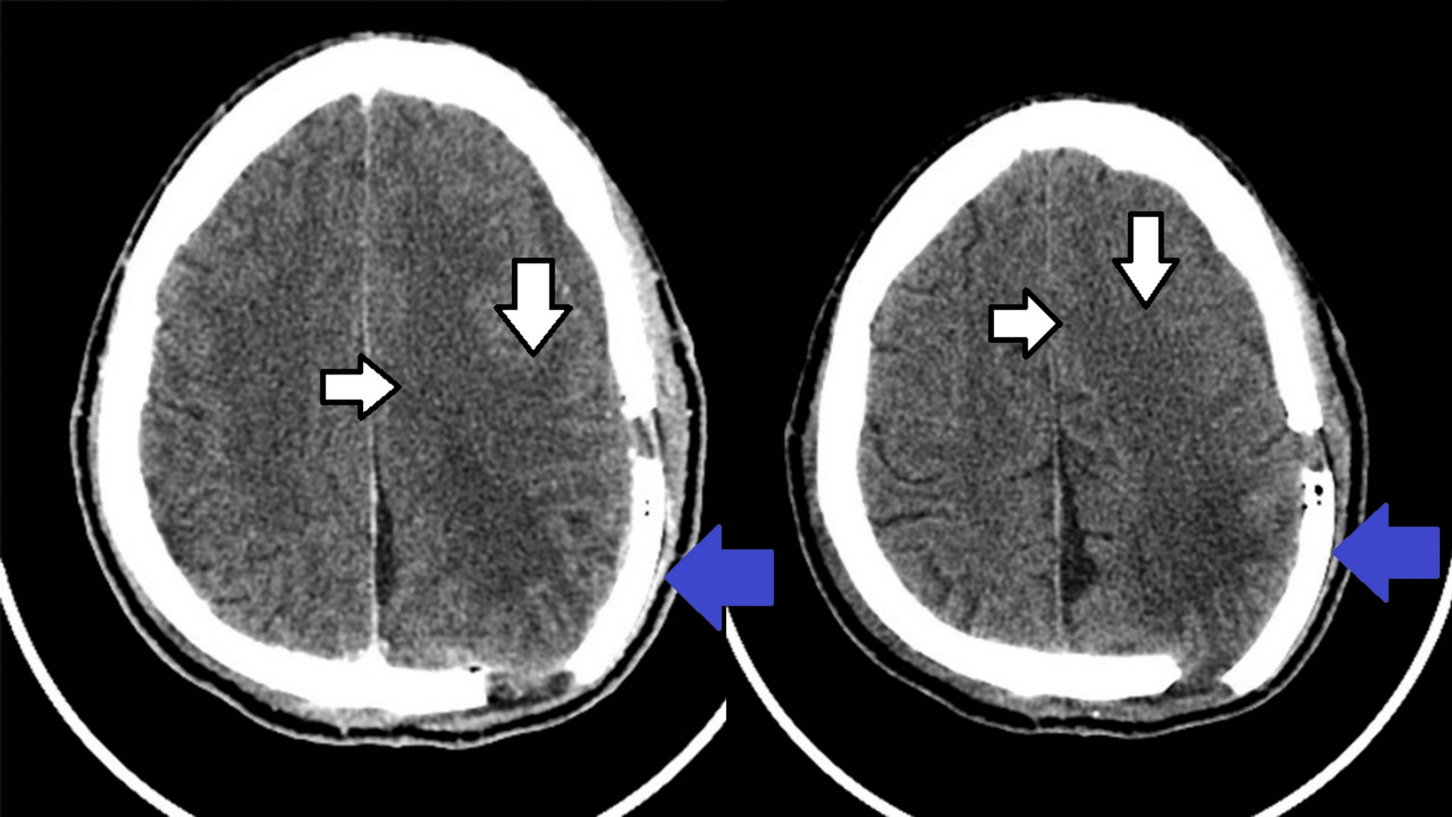
Non-contrast head CT scan showing the site of osteoplastic craniotomy (blue arrows) with gross total resection of the lesion and postoperative edema (white arrows with black outline).

Postoperative recovery was uneventful. The patient remained in the intensive care unit for 48 hours, where he received IV hydration with 0.9% saline, lansoprazole 30 mg daily, ceftriaxone 1 g twice daily, and levetiracetam 500 mg twice daily. His right-sided weakness gradually improved, and no further seizures were observed during hospitalization. He was discharged on postoperative day seven with oral levetiracetam, 500 mg twice daily. Postoperative CT confirmed complete tumor resection with no residual lesion (Figure 5).

This case highlights the clinical course of AM presenting with recurrent seizures and progressive focal neurological deficits. Despite the absence of MRI and cerebral angiography, a timely CT-based diagnosis, intraoperative recognition of a hypervascular lesion, and Simpson grade II resection resulted in complete tumor removal, seizure control, and neurological recovery. This underscores both the challenges faced by neurosurgical teams in resource-limited settings and the importance of careful surgical planning and hemostatic technique when preoperative vascular mapping is not available.

## Discussion

AM is a rare histological subtype of meningioma, representing approximately 2% of all cases [4]. It is classified as a WHO grade I tumor, a designation that reflects its generally benign biological behavior and favorable prognosis [5]. These tumors predominantly occur in adults, with a mean age of 50-64 years, and, interestingly, some series report a slight male predominance-contrasting with the female predominance usually observed in other meningioma subtypes [6].

Histologically, AM are characterized by a predominance of vascular components, with more than 50% of the tumor volume composed of blood vessels interspersed among meningothelial cells. Nuclear atypia and anaplasia are absent, and the proliferative index (Ki-67) is typically low (~2.4%), further supporting their indolent nature [7]. The most frequent anatomical sites include the cerebral convexities and parasagittal or falcine regions [8]. Clinical manifestations are determined largely by tumor size and location, with headaches, seizures, signs of intracranial hypertension, and focal neurological deficits being the most common presentations [9]. Convexity meningiomas, such as the parietal lesion in this case, have a particularly strong association with seizures due to their proximity to cortical structures. Postictal hemiparesis, although less common, can occur as a result of transient cortical dysfunction after generalized seizures [8]. In our patient, recurrent generalized tonic-clonic seizures with postictal hemiparesis were the main clinical manifestations that eventually prompted neurosurgical evaluation.

Radiologically, AM may mimic other hypervascular lesions, such as hemangioblastomas or hemangiopericytomas, because of their prominent vascularity [10]. This overlap underscores the importance of histopathological confirmation, which demonstrates meningothelial cells interspersed among abundant vascular channels. Immunohistochemistry, usually showing EMA and vimentin positivity, remains crucial for definitive diagnosis [1].

Although most meningiomas are effectively treated with surgical resection, published literature on AM remains limited to small case series and individual reports [8,9]. Gross total resection (Simpson grade I or II) is considered the treatment of choice and is associated with excellent outcomes [7]. Nevertheless, the prominent vascularity of these tumors introduces specific intraoperative and perioperative challenges. These include a higher risk of intraoperative hemorrhage, potential need for blood transfusion, obscured surgical planes that may hinder complete resection, and increased likelihood of cortical or venous injury. Additionally, the hypervascular nature of these tumors predisposes patients to postoperative hematoma formation, hemodynamic instability due to blood loss, and prolonged operative times [5,7]. In select cases, preoperative embolization may be considered to reduce intraoperative bleeding, though it carries inherent risks such as stroke or vessel perforation [5].

In our case, specific strategies were undertaken to mitigate these risks. Preoperative neuroimaging was carefully reviewed to delineate vascular supply and venous anatomy, facilitating differentiation from other hypervascular tumors. A wide craniotomy was performed to optimize exposure and venous control. Early coagulation of dural feeders and stepwise devascularization were employed before tumor debulking, effectively reducing intraoperative bleeding. Meticulous microsurgical dissection, combined with the use of adjunctive hemostatic agents, secured hemostasis throughout the procedure. Postoperatively, the patient was monitored in the intensive care unit to promptly identify potential complications, such as rebleeding, hematoma formation, or hemodynamic instability. These strategies allowed us to achieve gross total resection with minimal morbidity, aligning with the best practices reported in the literature [8].

This case contributes to the limited literature on AM by providing a comprehensive clinical, radiological, and histopathological description of this rare variant while illustrating the unique challenges of managing a hypervascular intracranial tumor in a resource-limited setting. Our experience underscores the importance of individualized surgical planning, careful intraoperative technique, and vigilant postoperative care in achieving favorable outcomes when preoperative vascular mapping is not available.

## Conclusions

AM is a rare but surgically curable tumor whose marked vascularity can present significant intraoperative challenges. This case illustrates that recurrent seizures with postictal deficits may be the first clue to its diagnosis and should prompt timely neuroimaging. In this setting, where MRI and cerebral angiography were unavailable, gross total resection was successfully achieved through careful CT-based planning, wide exposure, early devascularization, and meticulous microsurgical technique. The patient’s excellent postoperative recovery and seizure control highlight that, when advanced imaging is not accessible, tailored surgical strategies and intraoperative vigilance can still lead to favorable outcomes. This report reinforces the importance of anticipating vascular risks and adapting surgical planning to the available diagnostic resources.
